# Transcriptional response of *Methanosarcina acetivorans* to repression of the energy-conserving methanophenazine: CoM-CoB heterodisulfide reductase enzyme HdrED

**DOI:** 10.1128/spectrum.00957-24

**Published:** 2024-10-29

**Authors:** Nicole R. Buan, William W. Metcalf

**Affiliations:** 1 Department of Biochemistry, University of Nebraska-Lincoln, Lincoln, Nebraska, USA; 2 Department of Microbiology, University of Illinois, Urbana-Champaign, Urbana, Illinois, USA; University of Minnesota Twin Cities, St. Paul, Minnesota, USA

**Keywords:** methanogenesis, *Methanosarcina*, methanogens, archea, stress response, transcriptional regulation

## Abstract

Methane-producing archaea are key organisms in the anaerobic carbon cycle. These organisms, also called methanogens, grow by converting substrate to methane gas in a process called methanogenesis. Previous research showed that the reduction of the terminal electron acceptor is the rate-limiting step in methanogenesis by *Methanosarcina acetivorans*. In order to gain insight into how the cells sense and respond to the availability of the terminal electron acceptor, we designed an experiment to deplete cells of the essential terminal oxidase enzyme, HdrED. We found that the depletion of HdrED *in vivo* results in a higher abundance of transcripts for methyltransferases (*mtaC2, mtaB3, mtaC3*), coenzyme B biosynthesis, C1 metabolism, and pyrimidine compounds. In most cases, these changes were distinct from transcript abundance changes observed during the transition from exponential growth to stationary phase cultures. These data implicate the methylotrophic methanogenesis regulator MsrC (MA4383) in CoM-S-S-CoB heterodisulfide sensing and indicate cells have a specific mechanism to sense intracellular ratio of CoM-S-S-CoB, coenzyme M, and coenzyme B thiols and further suggest transcripts encoding translation and methanogenesis functions are controlled by feed-forward regulation depending on substrate availability.

**IMPORTANCE:**

*Methanosarcina* is an emerging model archaeon and synthetic biology platform for the production of renewable energy and sustainable chemicals to reduce dependence on petroleum. Research into metabolic networks and gene regulation in this organism and other methanogens will inform genome-scale metabolic modeling and microbial function prediction in uncultured or non-model anaerobes and archaea. This study suggests methanogens use unknown mechanisms to efficiently couple methanogenesis to gene regulation via CoM-S-S-CoB and ATP availability.

## INTRODUCTION

**Fig 1:**
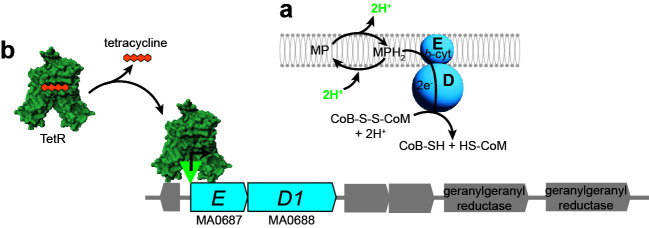
Role of HdrED in methanogenesis. Panel a, HdrED is a heme-containing membrane enzyme that conserves energy by oxidizing reduced methanophenazine (MPH_2_) and regenerating reduced coenzyme M and coenzyme B thiols (CoM-SH and CoB-SH, respectively). Panel b, strain WWM204 has had the native *P_hdrED_* promoter replaced by *P_mcrB(tetO1)_* promoter (green triangle). When tetracycline is present in the culture medium, the TetR repressor does not bind the *tetO1* operator, allowing *hdrED* expression. When tetracycline is removed, TetR binds *tetO1*, resulting in the repression of *hdrED* transcription.

Methanogens are archaea that conserve energy by producing methane gas in a process called methanogenesis. The membrane-bound cytochrome-containing coenzyme M–coenzyme B (CoM-S-S-CoB) heterodisulfide reductase enzyme is essential for methanogenesis by *Methanosarcina* species (1, 2). The membrane-bound heterodisulfide reductase HdrED functions to reduce CoM-S-S-CoB, thus regenerating thiol forms of coenzyme M (CoM-SH, mercaptoethane sulfonate) and coenzyme B (CoB-SH, 7-mercaptoheptanoylthreonine phosphate) for subsequent rounds of methanogenesis (Fig. 1) (1). HdrED uses reduced methanophenazine as an electron donor (3). Methanophenazine is a lipophilic membrane electron carrier functionally analogous to respiratory quinones found widely in other organisms (4). Upon oxidation of reduced methanophenazine by HdrED, protons are translocated across the cell membrane resulting in a proton gradient that is harnessed by ATP synthase for ATP production. Thus, HdrED serves a critical role in methanogen metabolism by conserving energy and acting as the terminal oxidoreductase in the electron transport chain of *Methanosarcina* species.

The deletion of non-energy-conserving cytoplasmic heterodisulfide reductase HdrABC enzyme results in higher metabolic efficiency but slower growth, and overexpressing HdrABC has the opposite effect (2). We wanted to further probe the system by asking what would happen if HdrED was depleted. What stress genes would be induced? Which metabolic genes would be repressed? Would other energy conservation or methanogenesis genes be upregulated?

Based on our current understanding of *M. acetivorans* metabolism, energy stress by depletion of HdrED should result in an immediate imbalance in CoM-S-S-CoB/CoM-SH + CoB SH metabolite pools and collapse of the transmembrane proton gradient. Thus, we would expect changes in gene expression that reflect a complex interplay between CoM-S-S-CoB and ATP concentrations in the cell (Fig. 1). Our initial hypothesis was that HdrED depletion may mimic the transition from exponential to stationary phase. However, *M. acetivorans*, like many archaea, do not have a bacterial-like stringent response, and there is limited information available on mechanisms of metabolic regulation in this organism. While *Methanosarcina* species have candidates for regulation by bacterial-type two-component regulators (5–7), small RNAs (8), histone-like proteins (9), and other mechanisms (10), mapping out the finer details of these gene regulatory networks is an exciting research area ripe for novel discovery.

In this study, we sought to gain insight into how *M. acetivorans* responds to energy stress by depleting cells of HdrED by transcriptional repression using a tetracycline-responsive TetR repressor (Fig. 1) (2, 11). The presence of tetracycline does not affect the growth of *M. acetivorans*, and the only gene shown to be differentially expressed due to exposure to tetracycline is a putative ABC transporter substrate-binding protein MA0887, making the TetR/Ptet system well suited to use for this purpose (12). We harvested mRNA from matched cultures in which inducer (tetracycline) was present or absent, and compared changes in transcript abundance between exponentially growing cells and cells in stationary phase cultures, and as *hdrED* transcription is repressed over time.

## MATERIALS AND METHODS

### Strains and culture conditions

*Methanosarcina* strain WWM204 (*Δhpt att::P_mcrB_-tetR/ϕC31 int/attB::hdrED::P_mcrB_-tetR frt/pac hpt/frt P_mcrB(tet01)_hdrED*) (2) was grown at 37°C under strictly anaerobic conditions in 10 mL HS mineral salts medium in 18 mm by 150 mm anaerobic culture tubes (13, 14). The following anaerobic additions were added when appropriate: MeOH (125 mM), tetracycline (100 µg/mL, +Tet), and puromycin (2 µg/mL). WWM204 was maintained with tetracycline at all times until commencing the experiment, and cultures were passaged fewer than 10 times before restarting from freezer stocks. In previous work, it was determined that, on HS methanol medium, the native *hdrED* promoter shows 401 ± 24 nmol min^−1^ mg^−1^ transcriptional fusion reporter activity (2), whereas the *P_mcrB(tet01)_* promoter has activity of <0.4 nmol min^−1^ mg^−1^ in the absence of inducer (−tet) and 792.8 ± 20.0 nmol min^−1^ mg^−1^ when inducer is added (+tet) (11). Growth was measured every 6 h by reading the optical density at 600 nm (OD_600_) in a Spectronic 21 spectrophotometer (Milton Roy Company).

### Transcriptomic data sets and statistical analysis

For total RNA extraction, WWM204 was grown on HS MeOH plus tetracycline. At mid-exponential phase (approximately OD_600_ 0.5), cells were anaerobically harvested by centrifugation in a clinical centrifuge and resuspended in 10 mL HS MeOH to remove excess tetracycline. Resuspended cells were inoculated 1:200 into fresh HS MeOH medium with tetracycline (one biological replicate) or without tetracycline (eight biological replicates). Cells were harvested by withdrawing 4 mL of culture anaerobically and directly lysing in 15 mL Qiagen RNEasy buffer RLT and frozen at −80°C until RNA extraction. For control (+Tet) samples, cells were harvested from the same culture at the exponential phase (OD_600_ 0.4) and stationary phase 24 h after reaching peak culture density (OD_600_ 0.725). Matched treatment samples (−Tet) were harvested between OD_600_ 0.3 and 0.5 every 6 h from independent biological replicates when the nominal specific growth rate began to diverge between the −Tet and +Tet cultures (Table 1). The nominal specific growth rate was determined by comparing the change in OD_600_ over the preceding 6 h period. A total of 17 RNA samples were collected. Total RNA was extracted using the Qiagen RNEasy midiprep kit, and DNA contamination was removed by digestion with TurboDNAfree DNAse (Ambion) and confirmed by lack of product after 40 cycles of PCR amplification.

**TABLE 1:** Experimental design for RNA sampling^*c*^

Treatment	Tube #	Time at sampling (h)	OD_600_ at sampling	Time at sampling (h)	OD_600_ at sampling
+Tet	1	24^*a*^	0.400	36^*a*^	0.725
−Tet	2	30	0.295	48	0.425
	3	30^*b*^	0.300	48^*b*^	0.450
	4	36	0.330	NS	NS
	5	36^*b*^	0.345	42	0.405
	6	24	0.260	36	0.365
	7	24	0.265	42	0.405
	8	30	0.295	48	0.455
	9	24	0.245	42	0.445

^*a*^ Two technical replicates.

^*b*^ Did not meet quality control after conversion to cDNA and omitted from further analysis.

^*c*^ NS, not sampled.

RNA quality was verified with an Agilent Bioanalyzer. Samples which did not meet quality control standards were dropped from further processing. High-quality RNA samples [A_260/280_ >1.8, A_260/230_ > 1.8, and RNA Integrity Number (RIN) >8] were reverse-transcribed to cDNA and processed according to Nimblegen chip manufacturer instructions. A total of 16 *Methanosarcina acetivorans* C2A genome oligo chip sets were used for data collection. Nucleic acid quality assessment, hybridization, and chip scanning were done at the University of Illinois W.M. Keck Center for Functional Genomics. Microarray data have been deposited at the National Center for Biotechnology Information Gene Expression Omnibus database (NCBI GEO, https://www.ncbi.nlm.nih.gov/geo/) under accession GSE263381.

### Bioinformatics and statistical tests

Transcriptomic data were analyzed according to Affymetrix chip manufacturer instructions using NimbleScan v2.5 software. For this experiment, each individual culture was considered a biological replicate, and cDNA hybridization arrays were considered technical replicates. Hybridization signal reads from four technical replicate arrays were normalized to the mean signal intensity, and log2 fold change was calculated between pairwise comparisons (15–17). To assess the distribution of signal intensities across +Tet technical replicates, reads for each gene were normalized against the average signal intensity ($\overset{-}{i}$) using the following equation: $f(x)=\log_{2}\left(\frac{x}{\bar{i}}\right)$. Paired, two-tailed *t*-test was used to calculate *P* values for each gene. A stringent false discovery rate (FDR) for pairwise comparisons was set to 0.01 to minimize the potential for false positives and differentially expressed genes (DEGs) were called using the Benjamini-Hochberg Procedure (18). Raw reads and differentially expressed genes (>log2 FC, FDR < 0.01) are provided in Supplementary Material. DEGs were analyzed using the Gene Ontology Resource (http://geneontology.org/) with FDR < 0.05 default settings (19). Pathway network analysis was done using the STRING database (https://string-db.org/) with default settings.

## RESULTS

### Repression of *hdrED* results in immediate changes in specific growth rate

**Fig 2:**
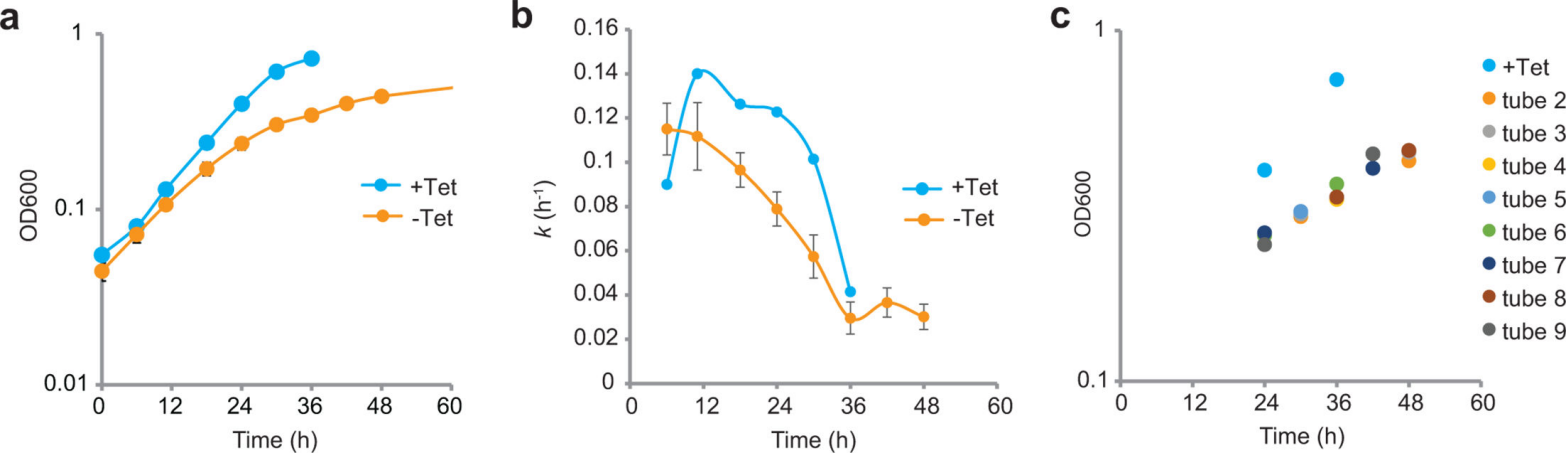
Sample preparation. Panel a, growth of *M. acetivorans P_tet_hdrED* on methanol with tetracycline (blue) and when inoculated into medium without tetracycline (orange). Panel b, nominal specific growth rates. Panel c, culture density vs time when samples were harvested. Error bars of −Tet samples indicate standard deviation but may be hidden behind symbols (*n* = 8 biological replicates). OD_600_, optical density at 600 nm; Tet, tetracycline.

To measure transcription dynamics in the *P_tet_hdrED* strain, a method was developed to rapidly dilute tetracycline inducer (Tet) from the culture medium and harvest RNA from matched cultures over time. RNA was harvested at 24 h after inoculation (Fig. 2a). Calculation of specific growth rate showed cultures with tetracycline (+Tet) maintained a high specific growth rate for 30 h and began to enter the stationary phase by 36 h after inoculation, while cultures without tetracycline (−Tet) steadily decreased growth rate over time (Fig. 2b). Every 6 h, samples were drawn from staggered cultures when OD_600_ was between 0.25 and 0.5 in the exponential phase (Table 1). For comparison, samples were taken from a culture in which tetracycline inducer was included when culture density was in the exponential phase (OD_600_ 0.4) and just as the culture entered the stationary phase at OD_600_ 0.725 (Fig. 2c). These data show that the depletion of the inducer results in the deceleration of growth within one generation, likely due to the repression of *hdrED* transcription. A similar effect is observed when the non-energy-conserving methylotrophic-specific CoM-S-S-CoB heterodisulfide reductase enzyme HdrABC is deleted and overexpressed; together these observations support the interpretation that metabolic flux through CoM-S-S-CoB is rate-limiting in *M. acetivorans* (2, 20).

### Distribution of RNA transcripts in exponentially growing vs stationary-phase cells

**Fig 3:**
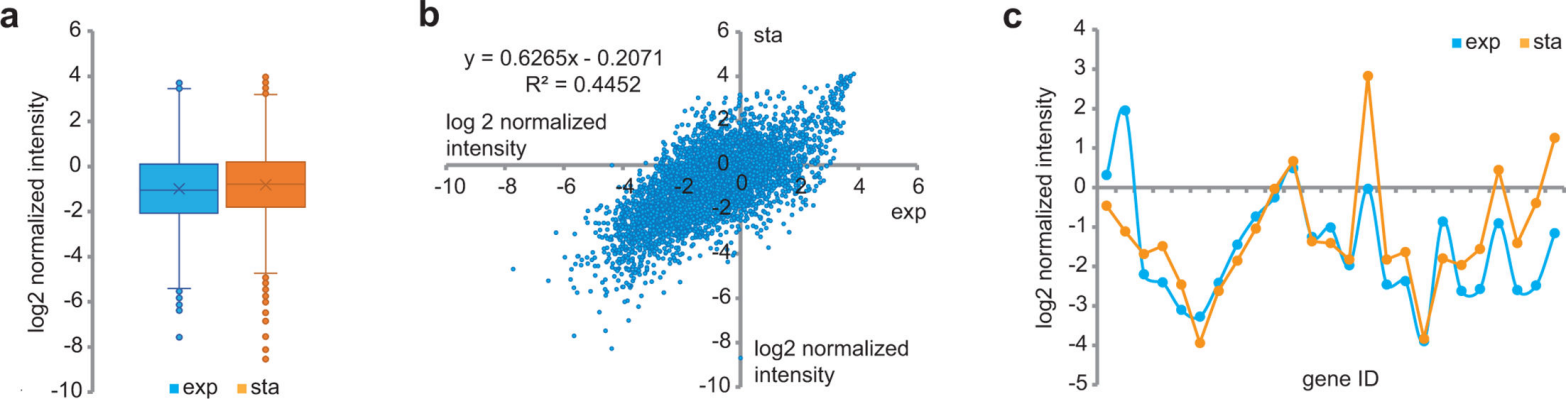
Differences in transcript abundance between exponentially growing cells vs cells entering stationary phase. Panel a, box plot of normalized transcript abundance in exponentially growing vs stationary phase cultures shows the uniform distribution. Panel b, correlation between transcript abundance during exponential growth vs stationary phase. Panel c, detail of transcript abundance for a sampling of 25 genes (MA0001, MA0010, MA1024-33, MA0101, MA1034, MA1039-44, MA1046, MA0102, MA1047, MA1049) showing differential abundance for some and constant expression for others (Table S1). exp, exponentially growing cells; sta, stationary phase cells. FDR < 0.01.

We compared transcript abundance between exponentially growing cells vs stationary phase cells to determine if genes identified as differentially expressed due to *hdrED* repression could be explained by slow growth (for example, due to signaling involved in sensing rate of substrate uptake or rate of cell division) (Fig. 3a). No statistically significant difference in a number of gene calls or distribution of mRNA abundance was observed in our samples between exponential growth and stationary phase (Fig. 3b). At the global level, there is only a 44.5% correlation between log2 FC values vs the normalized mean under each condition, which suggests that widespread global regulation is occurring for ~55.5% genes on a growth-phase-dependent manner and is in agreement with observations made by others (2, 21). Some suites of genes appear to be tightly co-regulated, as the relative mRNA abundance tracks tightly between the exponential and stationary phase (Fig. 3c). Overall, 1,952 genes were differentially regulated between exponential and stationary phase cultures (out of 4,433 annotated transcripts, 44.03%) (Table S1).

During the exponential phase, many hypothetical genes were highly expressed. Other abundant transcripts included DNA polymerase (MA0598) and ribosomal proteins. During the stationary phase, MA0687 *hdrE* is upregulated 1.37 log2 FC log_10_*P* = 4.94, but MA0688 *hdrD* is not differentially expressed. Transcripts for transposases (MA1019-1023, MA1029) amino acid permease (MA2426), and oligopeptide transporters (MA4253), among others, were also more highly expressed in the stationary phase.

### Global changes in transcript abundance upon repression of hdrED

**Fig 4:**
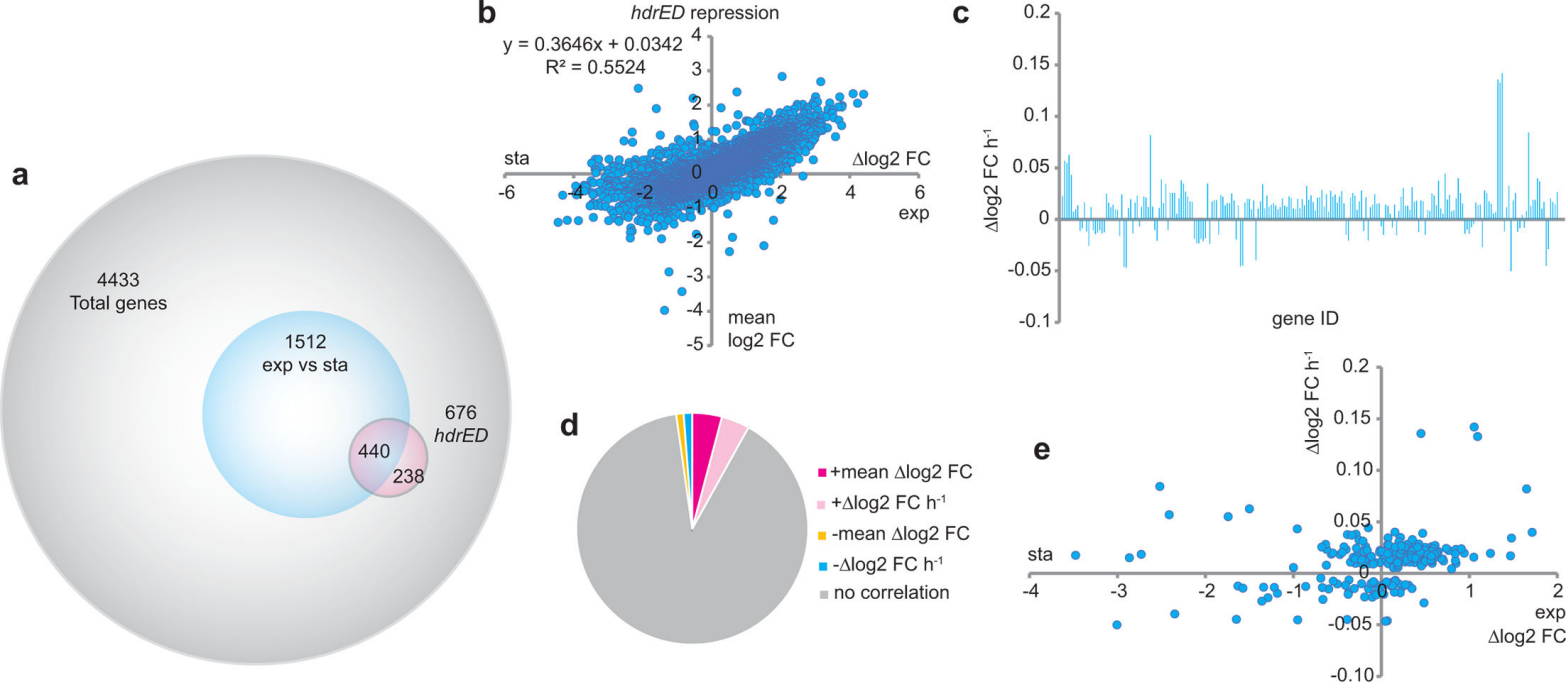
Analysis of transcript abundance changes due to repression of *hdrED*. Panel a, Venn diagram of total genes (gray), differentially expressed genes between exponentially growing vs stationary phase cultures (+Tet, blue), and differentially expressed genes when *hdrED* expression is repressed (−Tet, magenta). Panel b, correlation between average abundance of transcripts when *hdrED* is repressed over time vs +Tet exponentially growing cells plotted against the log2 FC between exponentially growing vs stationary phase cultures. Panel c, rate of change in transcript abundance due to repression of *hdrED* by genome position. Panel d, Proportion of genes that do not change transcript abundance due to *hdrED* repression (gray), average >log2 FC (magenta), increasing +Δlog2 FC h^−1^ (pink), decreasing −Δlog2 FC h^−1^ (cyan), and average −log2 FC (orange). Panel e, change in transcript abundance over time due to *hdrED* repression plotted against change in transcript abundance due to culture growth phase. False discovery rate is <0.01. Pearson correlation coefficient *r*^2^ >0.8. exp, exponentially growing cultures; sta, stationary phase cultures.

A subset of genes showed differential transcript abundance when strain WWM204 is inoculated into a medium without Tet. Of 4,433 genes, 676 showed changes in gene expression (Fig. 4a; Table S2). Of these 676, 440 were also regulated by the growth phase and showed changes between exponentially growing or stationary phase cells when averaged across all five time points (−Tet sampled at 24, 30, 36, 42, and 48 h). There was a 55% positive correlation between the growth phase and HdrED depletion suggesting tight coupling between HdrED-dependent gene regulation and general metabolic processes (Fig. 4b). This observation is consistent with current understanding as HdrED is thought to play an essential role in energy conservation and methanogenesis but also indicates some genes are independently controlled by HdrED. During the time course of the experiment, 227 genes showed time-dependent trends in transcript abundance with strong Pearson correlation coefficients (*r*^2^ >0.8) (Fig. 4c). Overall, of the 4,433 transcripts, 183 had increased abundance in HdrED depleted cells (mean log2 FC > 1) when averaged across all five time points, with another 174 transcripts displaying strong time-dependent increased abundance (*r*^2^ >0.8), 46 transcripts had decreased abundance in HdrED depleted cells when averaged over all five time points (mean log2 FC > 1) and 53 transcripts displayed strong time-dependent decreasing abundance (*r*^2^ >0.8) relative to the +Tet exponential control condition (Fig. 4d). Globally, the rate of change in transcript abundance over time was not correlated with the growth phase (Fig. 4e). This analysis is not to suggest that all possible temporal gene expression patterns were captured; rapid increase/decrease, cyclic or plateauing transcriptional responses may not have been observed between the sampling windows chosen in this experiment.

### Genes uniquely correlated with repression of hdrED

**Fig 5:**
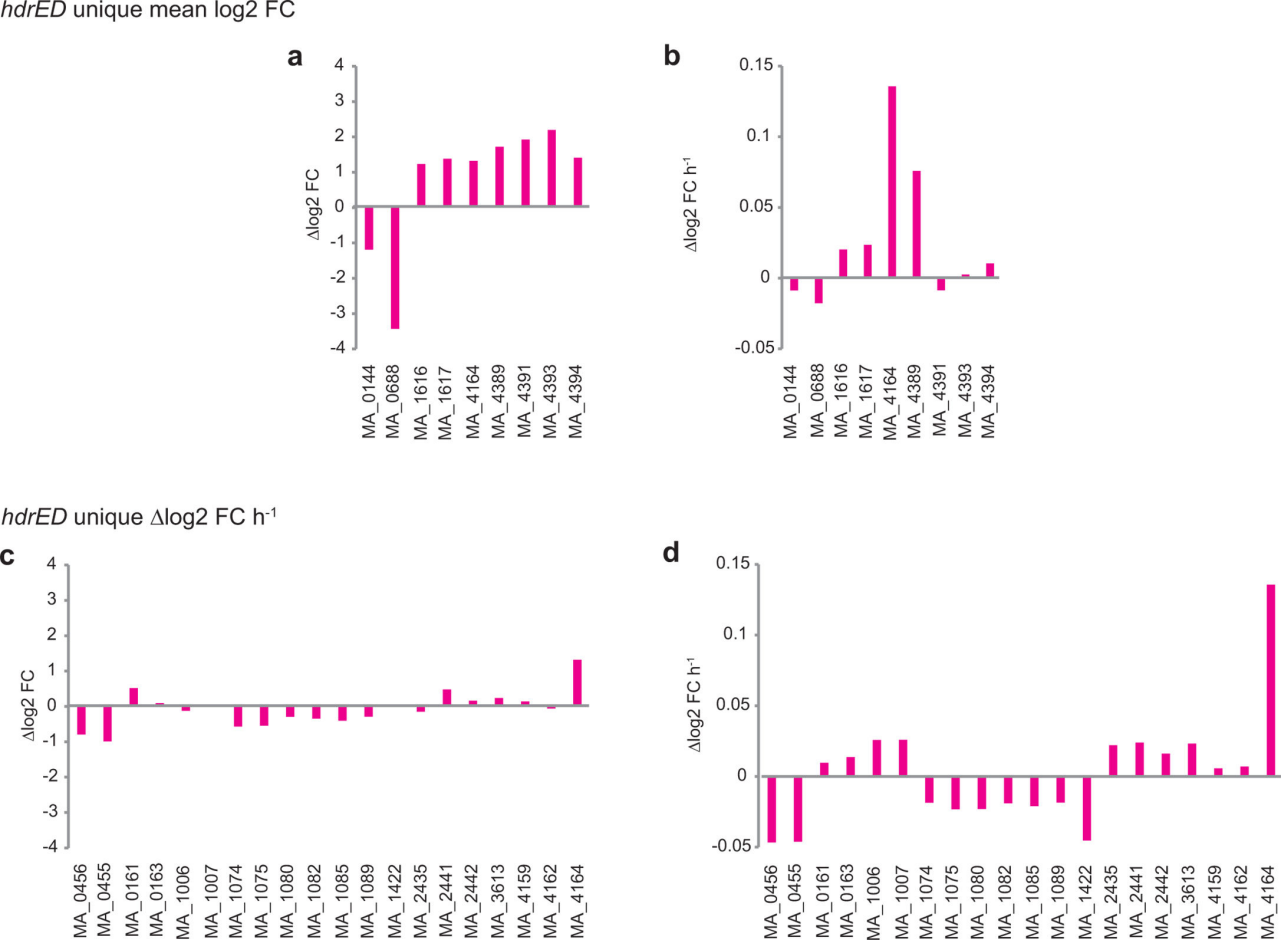
Unique changes in transcript abundance due to *hdrED* repression. Panels a and b, genes with increased average transcript abundance when *hdrED* is repressed but which did not change between exponentially growing vs stationary phase cells. Panels c and d, genes which showed a change in gene expression over time due to *hdrED* repression, but which did not change between exponentially growing vs stationary phase cells. Panels a and c, average Δlog2 fold change (FDR < 0.01). Panels b and d, calculated slopes. Data for selected genes are shown. A complete list of genes can be found in Table S2. Note MA_4164 is included in all panels as it met both statistical criteria.

To identify genes which are specifically related to repression of hdrED, transcripts were filtered according to whether they were stably expressed in both exponentially growing cultures vs stationary phase cultures, which were differentially expressed vs exponentially growing cultures when tetracycline was omitted (mean log2 FC > 1). Of 4,433 total transcripts, 13 genes were upregulated by *hdrED* repression and 14 genes were downregulated across all time points, including *hdrD* (MA0688, −3.4 log2 FC, log10 pval = 8.39) (Fig. 5a and b; Table S2). The transcripts with the largest increase in abundance when averaged across all five time points were hypothetical proteins (MA4565, MA2945, MA2324, MA1757), *mtaCB2* methanol corrinoid methyltransferases and nearby genes (MA4389, MA4391, MA4393, MA4394), *mtaCB3* methanol corrinoid methyltransferases (MA1617, MA1616), methylsulfide corrinoid protein *mtaC* MA4164, iron-sulfur flavoprotein MA1773, and coenzyme B biosynthesis gene 2-isopropyl malate synthase MA4615. In addition to *hdrD* MA0688, transcripts with decreased abundance when averaged across all five time points were hypothetical genes (MA4642, MA3475, MA3478, MA2690), monomethylamine methyltransferase *mtmC1* MA0144, succinoglycan biosynthesis regulator MA4199, phosphate ABC transporter permease MA0888, NADH dehydrogenase subunit N MA4368, fibrillarin MA0341 (pre-rRNA processing), 6-pyrovoyltetrahydropterin synthase MA4196, D-lactate dehydrogenase MA4631, chemotaxis signaling protein MA3070, and archaeosine tRNA ribosyltransferase MA4632. Another 159 transcripts had strong positive time-dependent abundance (*r*^2^ > 0.8), and 43 transcripts had strong time-dependent decreasing abundance (*r*^2^ > 0.8). The top 10 most significant increasing and decreasing time-dependent trends for genes with predicted functions are shown in Fig. 5c and d.

### Gene ontology and network analysis

**Fig 6:**
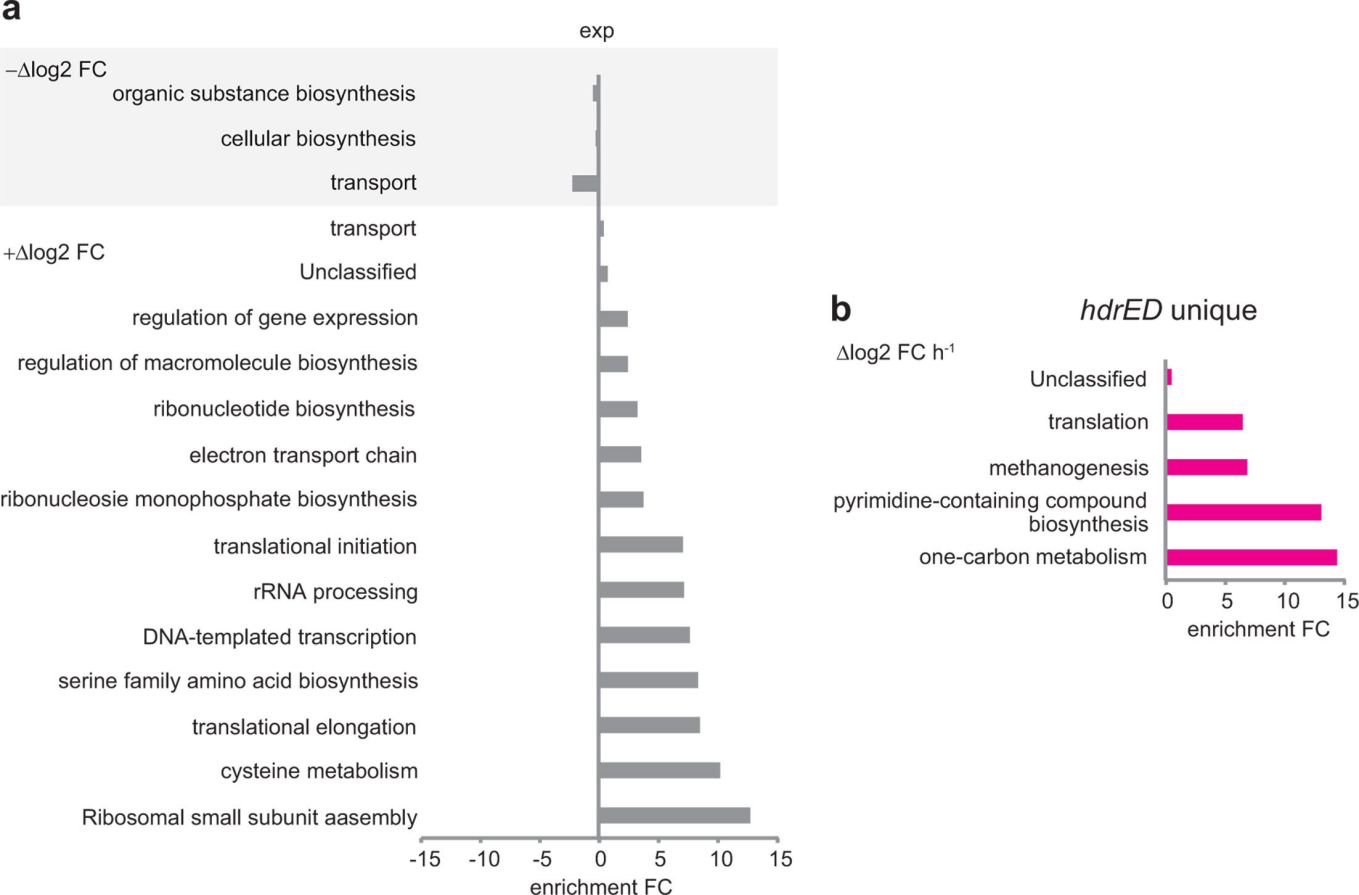
Gene ontology analysis. Panel a, Processes differentially regulated between exponentially growing cultures (exp) and stationary phase cultures (sta). Panel b, Processes differentially regulated due to *hdrED* repression. FDR < 0.05.

Differentially expressed genes were analyzed by gene ontology to identify trends in regulated pathways (Table S3). During exponential growth, a wide array of cellular processes are upregulated; most notably ribosome biogenesis, cysteine metabolism, DNA synthesis, and translation (Fig. 6a). In stationary phase cells, transporters and biosynthesis are downregulated. In comparison, when HdrED is repressed, transcripts for carbon metabolism, pyrimidine biosynthesis, methanogenesis, and translation trend toward repression, suggesting these mRNAs are less abundant or possibly less stable when the rate of energy conservation decreases due to HdrED depletion in cells (Fig. 6b).

**Fig 7:**
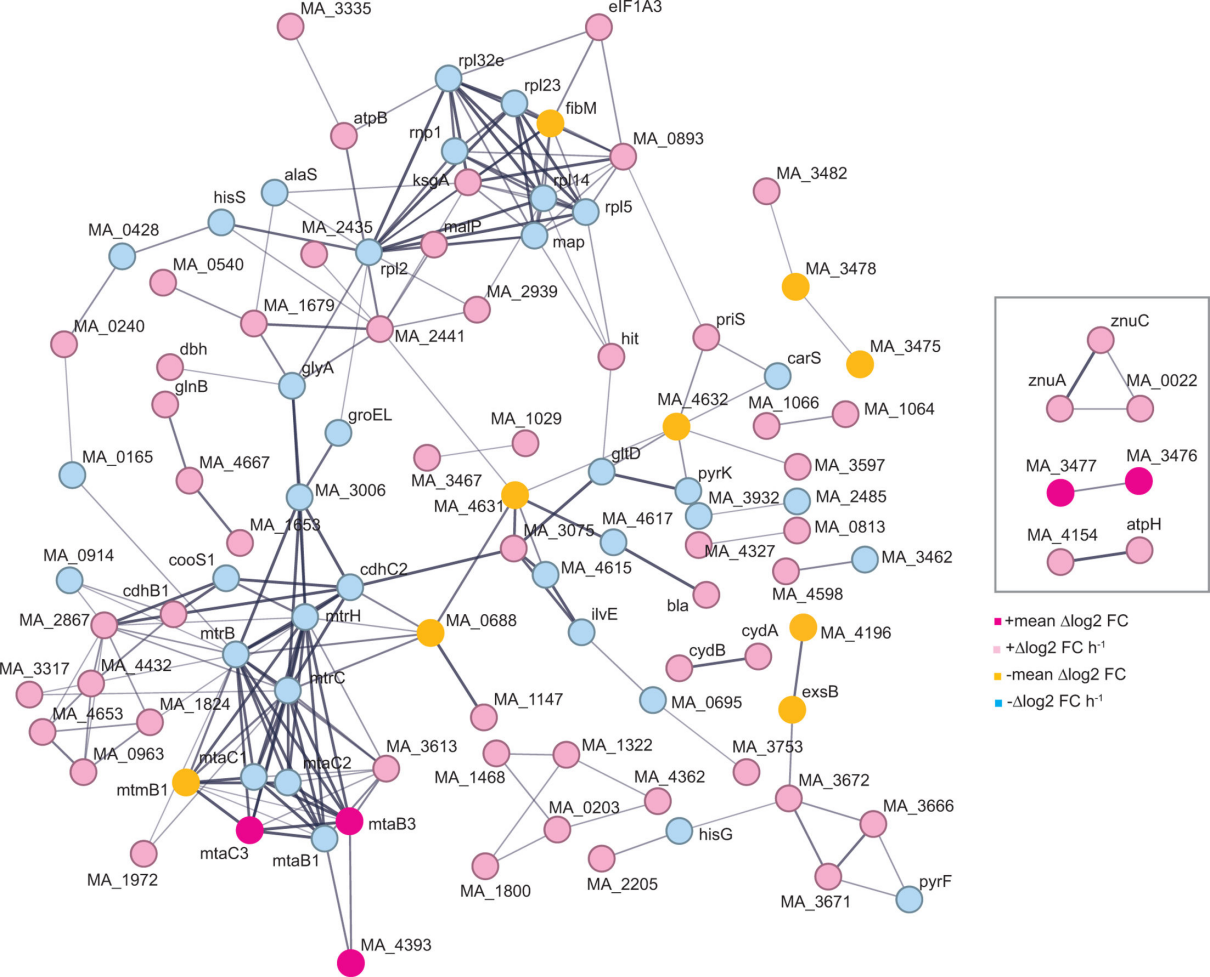
Interaction networks of genes regulated due to *hdrED* repression. Left panel, unique changes in transcript abundance due to *hdrED* repression. Inset, genes differentially expressed due to *hdrED* repression and exponential phase. Orphan nodes have been omitted.

To identify known interaction networks, differentially expressed genes were analyzed using the STRING database (Fig. 7). Network analysis revealed clusters of genes related to methylotrophic methanogenesis, ribosome biogenesis, and translation. The most cohesive network clusters show the depletion of HdrED results in the upregulation of ATP synthase, iron and zinc transport, and methanol methyltransferases MtaCB3, while decreased HdrED also results in the downregulation of transcripts for monomethylamine methyltransferase MtmB1, methanol methyltransferase MtaCB1, Mtr (CH_3_-S-CoM:tetrahydromethanopterin methyltransferase), and CooS (carbon monoxide dehydrogenase). These relationships suggest methanogenesis transcripts are directly and indirectly sensitive to CoM-S-S-CoB and ATP concentrations. While insight gleaned from network analysis in *M. acetivorans* is somewhat limited due to the large number of genes with unknown functions or general predicted function, it is clear that regulation patterns are highly complex, suggesting multiple interacting factors, metabolites, and transcription regulators affecting transcript abundance in cells.

**Fig 8:**
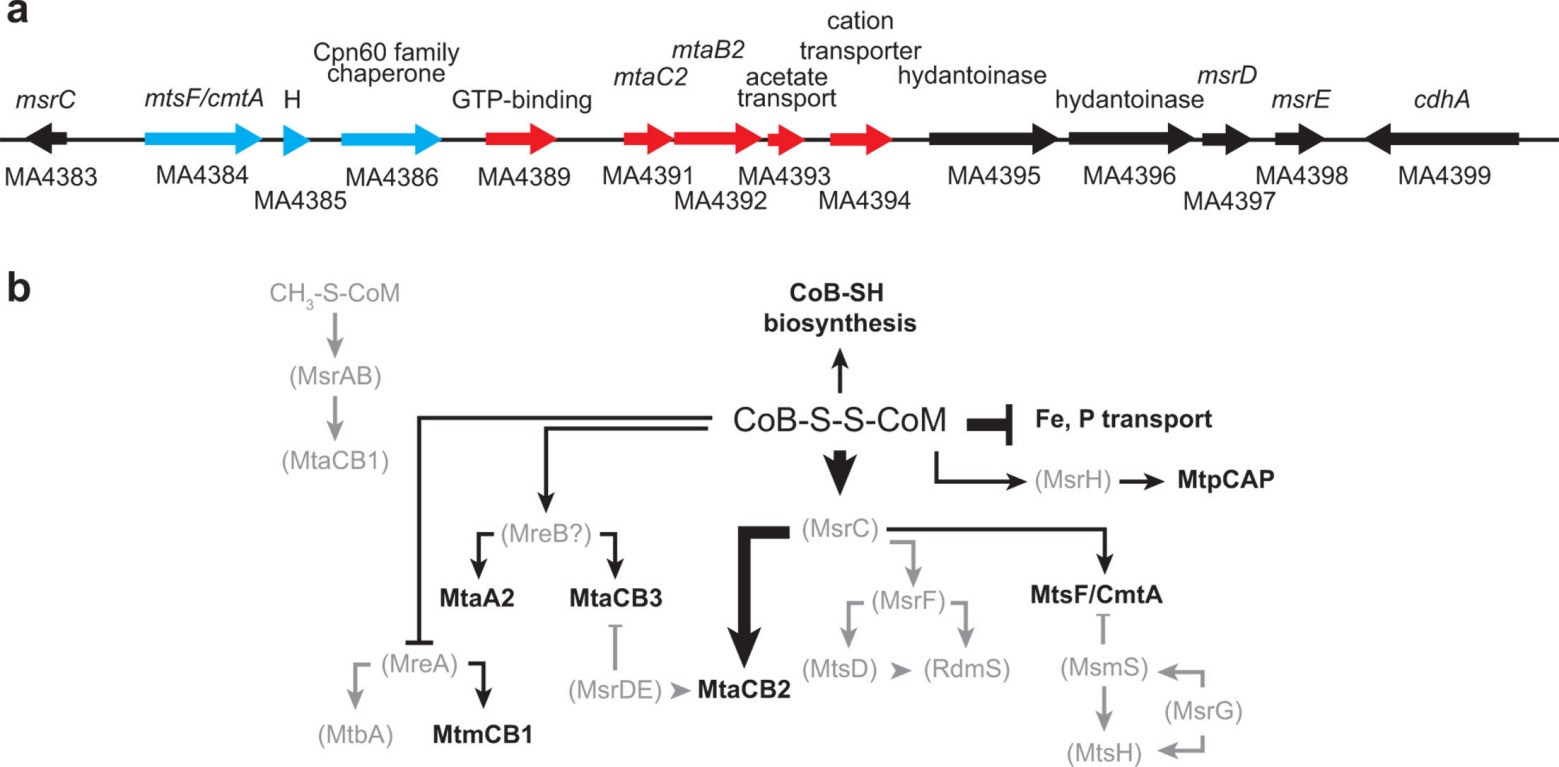
Putative model for CoM-S-S-CoB regulation network. Panel a, genomic region around the *mtaCB2* genes. Red arrows show transcripts upregulated when *hdrED* is repressed. Blue arrows show transcripts increased when *hdrED* is repressed and in stationary-phase cells. Panel b, known methylotrophic methanogenesis regulation network. Arrowheads indicate positive gene correlations. Tails represent negative correlations. Line thickness represents the relative effect on gene expression when HdrED is depleted. Grayscale indicates transcripts unaffected by transcriptional repression of *hdrED*.

By arranging differentially regulated genes in sequential genome order, it became apparent that the longest contiguous cluster of genes regulated by HdrED repression ranged from MA4384 to MA4394 (Fig. 8a). This region is directly downstream of the gene for the methylotrophic methanogenesis regulator *msrC* and encompasses genes for the fused corrinoid/methyltransferase protein *mtsF/cmtA* as well as the methanol:CoM methyltransferase and corrinoid protein *mtaCB2*. Downstream of these differentially regulated genes is predicted hydantoinases MA4395 and MA4396 as well as the *msrDE* regulator, which induces the expression of *mtaCB2* and represses *mtaCB3* methanol methyltransferase. Taken together with other differentially regulated genes, our observations suggest that HdrED depletion causes the cell to upregulate coenzyme B biosynthesis while repressing phosphate and iron transport, upregulating expression of genes controlled by *msrC*, *msrH* and possibly methylotrophic regulator *mreB* protein while upregulating *mtmCB1* monomethylamine:CoM methyltransferase, likely by inhibiting the methylotrophic regulator *mreA* (Fig. 8b). We propose that while phosphorelay likely plays a role in signal transduction and activation/repression of the transcription factors, we note that differential regulation of these genes was observed independently of growth rate under conditions when methanol substrate was abundant; these transcripts were differentially regulated only when depleted of HdrED and not when cells were in exponential vs stationary phase, suggesting that CoM-S-S-CoB itself or severe lack of CoM-SH or ATP was necessary to induce transcriptional effects.

## DISCUSSION

**Fig 9:**
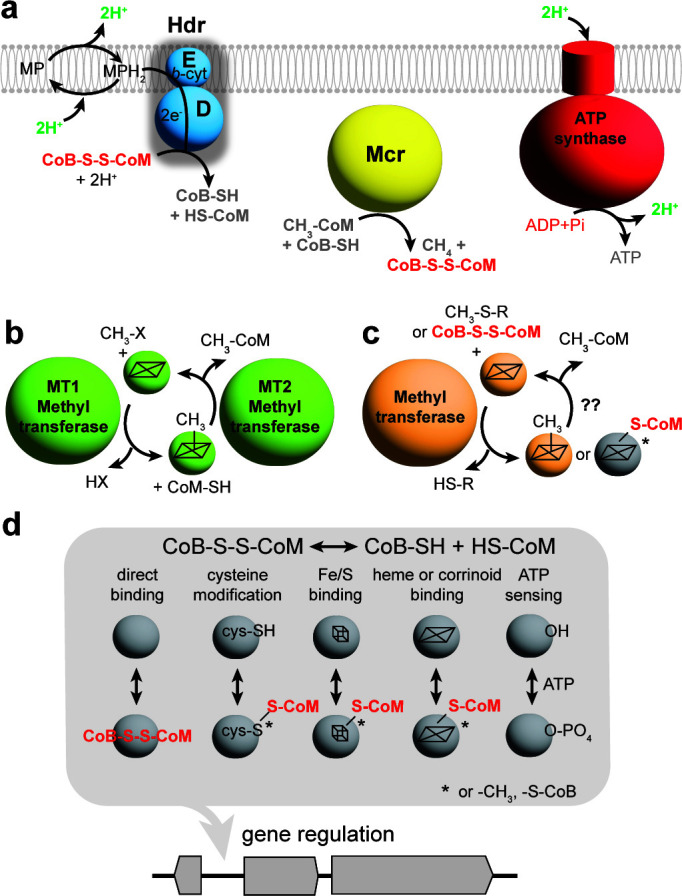
Postulated effect of depleted HdrED in *M. acetivorans*. Panel a, depletion of HdrED (blue complex) limits the rate of reduction of CoM-S-S-CoB to CoM-SH and CoB-SH as well as decreased generation of transmembrane proton gradient. Decreased CoM-SH results in less conversion of CH_3_OH substrate to CH_3_-S-CoM, and thus decreased substrate concentration for methyl-coenzyme M reductase (Mcr, yellow). Decreased translocation of protons by HdrED results in less ATP production by ATP synthase (red complex). Panel b, MT1 methanol:corrinoid methyltransferases and MT2 methylcorrinoid:CoM-SH methyltransferases are upregulated by HdrED depletion (green) as well as methylthio methyltransferases (orange, Panel c). Panel d, possible direct and indirect mechanisms for sensing changes in CoM-S-S-CoB/CoM-SH + CoB SH enzyme activity. CH_3_-X, methylated substrate, HX, demethylated substrate; CH_3_-S-R, methylthio substrate; HS-R, demethylated methylthiosubstrate; MP, oxidized methanophenazine; MPH_2_, reduced methanophenazine.

*M. acetivorans* is an emerging model methanogen with potential to be used for the sustainable production of methane and other chemicals (22). Understanding how the organism senses changes in metabolic flux to balance growth vs methane production or to respond to stress is a critical knowledge gap that must be filled to improve metabolic engineering efforts. Our study suggests *M. acetivorans* directly and indirectly senses CoM-S-S-CoB accumulation in cells and responds by upregulating corrinoid methyltransferases, particularly MtaCB2 and MtaCB3, MtsF/CmtA and MtpCAP (Fig. 9a). CoB-SH biosynthesis is also upregulated by an unknown sensor and transcription factor while iron and phosphate transport repressed, presumably because without the ability to synthesize ATP, cells have abundant iron, a major constituent of iron-sulfur clusters used for enzyme cofactors, and are replete with phosphate. We assume HdrED depletion results in increased CoM-S-S-CoB and/or decreased CoB-SH concentration, while CoM would be primarily methylated as CH_3_-S-CoM. As a result, methyl-coenzyme M reductase (Mcr) activity would decrease, changing ratios of oxidized/reduced electron carriers, and ultimately decreasing ATP synthesis (Fig. 9a). Methyltransferase gene expression may be upregulated to increase the flux of substrate uptake to generate more CH_3_-S-CoM (Fig. 9b) and/or potentially by nonspecifically mimicking alternative methylated thiol substrates, for example, methylmercaptopropionate (Fig. 9c) (23). Changes to ratios of CH_3_-S-CoM/CoM-SH, oxidized/reduced electron carriers, or ATP/ADP may directly or indirectly affect gene expression; however, experimental evidence is yet to be obtained for how this would occur. Direct substrate binding, thiol-dependent binding at cysteine residues, iron-sulfur-clusters, or tetrapyrroles (heme or corrinoid cofactors), as well as phosphorelay are likely possibilities for how HdrED depletion may affect gene expression (Fig. 9d). At this time, not enough is known, in general, about gene regulatory mechanisms and the role of post-translational modifications in gene expression in *M. acetivorans* to definitively propose a molecular mechanism for how HdrED depletion could be directly or indirectly affecting gene expression.

Our observations suggest MsrC, although not itself differentially expressed, plays a major role in responding to the accumulation of CoM-S-S-CoB, either directly by binding CoM-S-S-CoB or indirectly through an unknown mechanism (Fig. 9d) (6). Likewise MsrH, MreA, and potentially MreB are involved in priming metabolism for methylotrophic methanogenesis from an alternate substrate (such as acetate, methylamines, or methylated sulfur compounds) (24, 25). Although in this experiment methanol substrate was not limiting, MreA, MreB, and MsrH appear to be involved in the response to accumulation of CoM-S-S-CoB. MreA and MreB are involved in regulating the switch from methanol to acetate, while MsrH regulates the expression of methyltransferases involved in the metabolism of methylated sulfur compounds (24, 25). Mtr CH_3_-CoM:H_4_MPT methyltransferase was also differentially regulated, mimicking the downregulation of the oxidative branch of methanogenesis observed when cells switch from methanol to acetate via MreA (25). However, it appears as if other methylotrophic and aceticlastic methanogenesis regulators MsrAB, MsrDE, MsrF, MsrG, MtsD, MtsH, MsmS, or RdmS are not involved in responding to energy stress caused by HdrED depletion. This may be because these other regulators require a signal that is absent or unaffected by HdrED activity.

In previous studies, the deletion of the nonessential methylotrophic ferredoxin:CoM-S-S-CoB heterodisulfide reductase HdrABC (*ΔhdrABC*) resulted in upregulation of genes for coenzyme M and coenzyme B biosynthesis as well as genes for MtsD, MtsH, MtsF/CmtA, and MtpC and MtpA involved in regulation and methyl transfer for methanogenesis from methyl sulfide and methylated sulfur compounds (2). Many of these same genes, but not all, are upregulated when HdrED is depleted. Together, the data paint a picture, whereby the depletion of HdrED results in metabolic changes, possibly a slight increase in CoM-S-S-CoB, which cells respond to by upregulating MtaCB2 and MtsF/CmtA via MsrC, by adjusting Fe and P transport, and by upregulating CoB-SH biosynthesis through unknown regulators. Secondarily, MtmBC1, MtaA2, MtaCB3, and MtpCAP methyltransferase systems are upregulated via MreA, MreB, and MsrH, respectively. When the rate of CoM-S-S-CoB reduction is chronically low, as it is in the *ΔhdrABC* deletion mutant, the cell adjusts CoM-SH, CoB-SH, and CoM-S-S-CoB homeostasis by upregulating CoM biosynthesis and by activating MtsD and MtsH (2). Overexpression of MtsF/CmtA and MtpCAP then allows cells to regenerate CoM-SH from CH_3_-S-CoM to produce methylsulfide (MeSH) or dimethylsulfide (DMS), which can be re-metabolized (2). Increased expression of *mtpCAP* was also observed when cells are grown on CO (26–28), when methyl-coenzyme M reductase enzyme is depleted in cells (12), and when the pyrollysine tRNA is deleted (29). This MeSH/DMS bypass is accelerated when sulfide concentrations are high, or when acetate and CoM-SH are supplemented to the culture medium (20).

There were other broad insights into *M. acetivorans* gene regulation that can be inferred from the study. While ribosomal protein genes were differentially regulated upon HdrED depletion, the magnitude of regulation was not higher than when comparing expression in exponentially growing vs stationary phase cells, suggesting that CoM-S-S-CoB is not a direct signal. *M. acetivorans* ribosome biogenesis is correlated with growth rate, similar to *E. coli*, and while the extent of the mechanistic similarities are not known, it is reasonable to suggest that *M. acetivorans* ribosome biogenesis may be sensitively regulated by ATP and the initiating nucleotide. Our results support this suggestion, as ribosome genes were upregulated somewhat when HdrED was depleted, albeit not drastically. We also observed that ATP synthases (MA2435, MA2441, and MA2442; MA4159) were upregulated, supporting the interpretation that HdrED is a major mechanism for energy conservation, and strengthening the concept that the rate of CoM-S-S-CoB reduction is very closely coupled to ATP synthesis. Intriguingly, cytochrome *d* methanophenazine oxidase (annotated as “cytochrome *d* ubiquinol oxidase”) *cydAB* (MA1006 and MA1007) showed increased transcript abundance over time. However, transcripts for other oxidative stress genes were not differentially expressed. CydAB was previously shown to be upregulated with exposure to O_2_ and was demonstrated to be used in conjunction D-lactate dehydrogenase (LDH, MA4631) to detoxify O_2_ (30). However, when HdrED is depleted, MA4631 is downregulated, indicating that CydAB and LDH can be regulated independently and that CydAB may be induced by an unknown mechanism directly by CoM-S-S-CoB or indirectly via a redox-sensing regulator, of which several have been characterized in *M. acetivorans* (5, 7, 11).

In conclusion, this study builds on the growing body of research into thiol and redox-dependent signaling networks in *M. acetivorans* and supports the central role of HdrED in energy conservation in this organism. Furthermore, it is clear that MsrC is emerging as a major regulator controlling a hierarchy of methylotrophic corrinoid methyltransferases. Our experimental design took great care to isolate the effect of CoM-S-S-CoB as much as technically possible, and the data presented here, when synthesized with available evidence, support the interpretation that cells directly sense CoM-S-S-CoB through an unknown biochemical mechanism, which could include direct binding by ionic interactions or by covalent binding and modification via a heme/corrinoid or surface cysteine as has been proposed for MsmS, RdmS, and MA4376 (Fig. 9d) (7, 31, 32).

## Data Availability

All data are provided in this manuscript and in Supplementary Information.

## SUPPLEMENTAL MATERIAL

Table S1 spectrum.00957-24-s0001.xlsx Microarray data: exponential growth vs stationary phase cultures.

Table S2 spectrum.00957-24-s0002.xlsx mRNA abundance from WWM204 -tetracycline cultures on HS MeOH. HdrED transcription is being repressed by TetR.

Table S3 spectrum.00957-24-s0003.xlsx GO enrichment analysis.

ASM does not own the copyrights to Supplemental Material that may be linked to, or accessed through, an article. The authors have granted ASM a non-exclusive, world-wide license to publish the Supplemental Material files. Please contact the corresponding author directly for reuse.
